# Discovery of Novel Materials with Broad Resistance to Bacterial Attachment Using Combinatorial Polymer Microarrays

**DOI:** 10.1002/adma.201204936

**Published:** 2013-02-18

**Authors:** Andrew L Hook, Chien-Yi Chang, Jing Yang, Steve Atkinson, Robert Langer, Daniel G Anderson, Martyn C Davies, Paul Williams, Morgan R Alexander

**Affiliations:** Laboratory of Biophysics and Surface Analysis, School of Pharmacy, University of NottinghamNottingham, NG72RD, UK; School of Molecular Medical Sciences, University of NottinghamNottingham, NG72RD, UK; Department of Chemical Engineering, Harvard-MIT Division of Health Sciences and Technology, David H. Koch Institute for Integrative Cancer Research, Massachusetts Institute of Technology500 Main Street, Cambridge, MA 02139, USA

Clinicians routinely have little choice but to employ materials known to support bacterial colonization in patients. The result is a high incidence of medical device-centred infections with increased morbidity and mortality in healthcare systems. Most healthcare-associated infections are associated with biofilms which form on the surfaces of medical devices.^1^ These are surface associated microbial communities, ‘slime cities’ within which bacteria acquire up to 1000 times higher tolerance to antibiotic treatment and the host immune system compared with their planktonic counterparts.^1^–^2^ The development of new materials that resist bacterial attachment and biofilm formation would offer significant therapeutic benefits and concomitant cost savings. Most strategies for reducing biofilm-associated infections focus on the modification of existing materials used to manufacture indwelling medical devices with compounds which kill bacteria, such as silver sulfadiazine, quaternary ammoniums, chlorhexidine, minocycline and rifampin.^3^–^6^ Greater success in preventing medical device-associated infections could be achieved by materials exhibiting inherent resistance to bacterial attachment and subsequent biofilm formation, as has been achieved using poly(ethylene glycol) brushes,^7^–^8^ and zwitterionic polymers.^9^–^10^ The discovery of new materials resistant to bacterial attachment is limited by the current poor understanding of bacterial response to materials.

Recently, a high throughput assay was developed that allowed the attachment of bacteria to be assessed on hundreds of unique acrylate and methacrylate polymers in parallel.^11^, ^12^ Using this platform a new class of materials was discovered with broad spectrum resistance to bacterial attachment.^12^ A total of 22 (meth)acrylate monomers were used to generate a library of 496 unique materials that were further evolved into lead materials. However, in excess of 100 (meth)acrylate monomers are commercially available and could be used to broaden the chemical diversity of the polymeric library used for screening.

In the present study a wider range of unique (meth)acrylate monomers (116) was used to screen for materials resistant to bacterial attachment. This exploration, comprising 1273 unique polymers in more than 10 000 separate assays, represents an exhaustive screen of the (meth)acrylate combinatorial space accessible with currently available off-the-shelf monomers. We used the multiple generation approach for screening,^13^ as depicted in **Figure** **1**a, where the lead materials evolve from first identification of homopolymers to co-polymerization and finally lead composition optimization. A first generation array composed of 4 repeats of 116 homopolymers was printed onto a poly(hydroxyl ethyl methacrylate) (pHEMA) coated glass slide (Figure 1a(i)). The pHEMA coating acted both as a low-fouling background and as an adhesion layer for the printed polymer spots.^14^ As a screen to identify materials with broad spectrum resistance to bacterial attachment, the polymer microarray were incubated with three different green fluorescent protein (GFP)-labelled bacterial species, *Pseudomonas aeruginosa* PA01, *Staphylococcus aureus* 8325-4 and uropathogenic *Escherichia coli* O6:K15:H31 (UPEC) for 72 h. After incubation the fluorescence due to each strain was quantified, normalized to the maximum level observed within the library for each strain, and averaged for each polymer to provide a measure of each polymer's bacterial performance *iota* (*í*) across all three strains using Equation 1. A complete list of the bacterial attachment to all 116 materials is provided in the Supporting Information (Table SI1). This measure was used to identify the top 18 materials with broad spectrum resistance to bacterial attachment, which included the 4 bacteria-resistant monomers identified in a previous screen.^12^ A total of 5 of these hit monomers contained fluorocarbon pendant groups, whilst 9 monomers included cyclic or aromatic hydrocarbon pendant groups. These monomers are shown in Figure 1b(i). In our previous study^12^ high throughput surface characterization^15^ implicated a number of polymer moieties in bacterial attachment. Specifically, hydroxyl groups were found to promote bacterial attachment whilst the combination of polar ester groups with hydrophobic pendant cyclic hydrocarbon groups was shown to resist bacterial colonization. All of the hit monomers discovered in this study produce polymers with amphiphilic pendant groups, consistent with this model.

**Figure 1:**
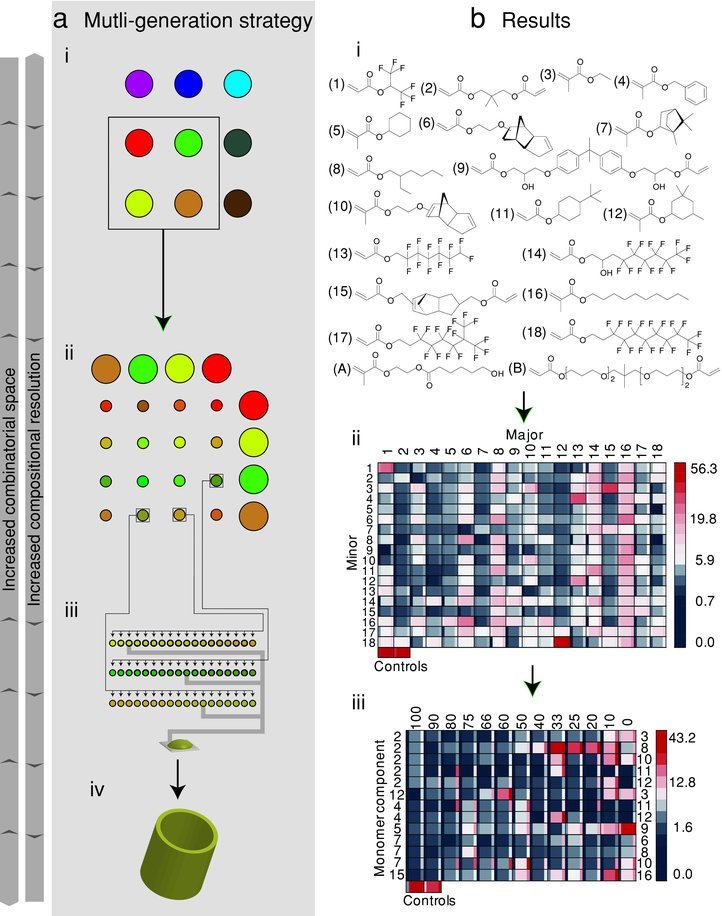
a) Schematic of the strategy applied to microarray formation. Initial arrays included a greater combinatorial space whilst latter arrays increased the compositional resolution at which the combinatorial space was explored. i) The 1st generation array consisted of 116 homopolymers, 18 of which were selected as “hits”. ii) The 2nd generation array consisted of 324 copolymers, formed by mixing 18 “hit” monomers pairwise. iii) The 3rd generation array explored 13 “hit” compositions from the 2nd generation array by incremental compositional variations. iv) The lead compositions from the 3rd generation were selected for scale-up and additional testing. b) Results from applying the microarray strategy. i) Chemical structures of hit monomers selected from the 1st generation array. ii) Intensity scale image of í/% for each of the materials in the second generation array, according to the scale given on the right. The scale is non-linear to highlight the range of the array. The materials were composed of two monomers mixed at a 2:1 ratio. The monomer used as the major or minor component is indicated across the first row or column respectively, and corresponds to the monomers in (i). The central square is the í, whilst the narrow columns to the left or right indicate ± one standard deviation unit, *n* = 3. iii) Intensity scale image of í/% for each of the materials in the third generation array, according to the scale given on the right. The scale is non-linear to highlight the range of the array. The monomers used are indicated to the left and right of the intensity scale, and refer to the monomers shown in (i). The content% of the monomers listed on the left is indicated in the top row. The central square is the í, whilst the narrow columns to the left and right indicate ± one standard deviation unit, *n* = 4. The controls were homopolymers of monomers A and B, which exhibited high bacterial attachment in the first generation array screen.


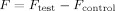
(1)

The 18 “hit” monomers were mixed pairwise at a ratio of 2:1 to create a second generation polymer microarray (Figure 1a(ii)) containing 324 unique materials. In particular, the second generation array was used to screen for synergistic effects caused by mixing the hit monomers since we have previously observed cases where a copolymer exhibited a biological performance that exceeds the performance of the respective homopolymers.^12^, ^16^ The *í* value for each of the 330 materials is shown in Figure 1b(ii). Generally lower bacterial attachment was observed on compositions containing hydrocarbon structures compared with materials containing fluorocarbons. From the second generation array the top 13 compositions with the lowest overall *í* were selected for use in a third generation array, listed in Figure 1b(iii). The focus of this array was to optimize the composition of the material. Thus, each composition was systematically varied between the ratios of 1:0, 9:1, 4:1, 3:1, 2:1, 1:1, 1:2, 1:3, 1:4, 1:9 and 0:1. This resulted in the formation of 169 unique materials. For comparative purposes, 2 positive controls which attracted high levels of bacterial attachment were also included in this array (monomers A and B in Figure 1b(i)). This third generation array was incubated with *P. aeruginosa*, *S. aureus* and UPEC for 72 h, and *í* was determined for each material. These results are summarized in Figure 1b(iii). The top 10 hit materials with the lowest observed bacterial attachment for all three strains were selected for further study. Within these 10 formulations, monomers 2, 11 and 12 (Figure 1b(i)) featured most frequently.

The final test of the efficacy of the hit materials was achieved by scaling up the hit formulations to 6–10 mm diameter polymer coupons. In this case, after incubation with *P. aeruginosa*, *S. aureus* and UPEC for 72 h the bacteria were stained with the DNA-binding dye (SYTO 17) and imaged by confocal microscopy for the determination of the area coverage of bacteria (%) on the polymer coupons. The resultant measured coverage is shown for each strain in **Figure** **2**b. Reference materials glass, TCPS and Bardex Bactiguard silver-containing hydrogel (a commerically available material for preventing device associated infections) were also assessed.^17^ Reduced bacterial coverage was measured for all hit materials for *S. aureus* and UPEC and for 6 of the 10 hit formulations for *P. aeruginosa* compared with the silver hydrogel. The material that performed best for each species was the homopolymer of monomer 7, which had a *P. aeruginosa* coverage of 3.7% ± 0.5% (1 standard deviation unit, *n* = 3), the homopolymer of monomer 15, which had a *S. aureus* coverage of 0.1% ± 0.03%, and the copolymer of monomer 7 (80% v/v) and monomer 6 (20% v/v), which had a UPEC coverage of <0.1% ± 0.02%. This corresponded to a reduction in bacterial coverage compared to the silver containing hydrogel of 81.4%, 99.1% and 99.3% for *P. aeruginosa*, *S. aureus* and UPEC, respectively. The material with the best broad spectrum performance was the homopolymer of monomer 15. Thus, the methodology described successfully identified materials that maintained their biological performance once scaled up. A large difference (>4×) in *í* measured on the microarray and on scaled up samples was observed for 4 of the 18 materials tested (Supporting Information, Figure SI2), which can be explained by altered surface chemistry driven by a larger surface area:volume ratio upon miniaturization.^12^

**Figure 2:**
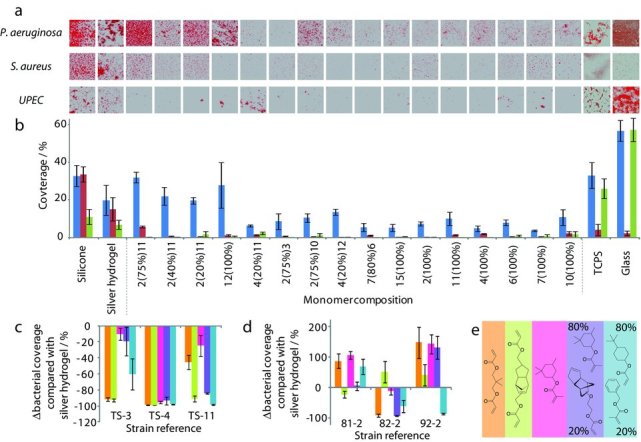
a) Confocal microscopy images of *P. aeruginosa*, *S. aureus* and UPEC stained with SYTO17 growing on polymer coupons and control materials. The identity of each material is shown in (b). Each image is 160 μm × 160 μm. b) Material scale-up: coverage of *P. aeruginosa* (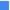), *S. aureus* (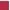), and UPEC (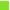) on polymer coupons of hit formulations. The error bars equal ± one standard deviation unit, *n* = 3. c,d) The Δ bacterial coverage on lead formulations compared with silver hydrogel for clinical strains of (c) *S. aureus* and (d) *P. aeruginosa*. The error bars equal ± one standard deviation unit, *n* = 5. The absolute values of the bacterial coverage are shown in Figure SI3 in the Supporting Information. e) Chemical structures of lead formulations color coded with reference to (c,d).

An important aspect of any prospective material for clinical application is the ability to resist not only laboratory-adapted bacterial strains but also fresh clinical isolates. We therefore selected the material with the lowest í in scale out, the material with the lowest *í* in scale-up, and the 3 materials with the lowest *F*_PA_, *F*_SA_ or *F*_UPEC_ in scale-up as 5 lead candidates resistant to bacterial attachment. These materials were incubated with clinical isolates of *S. aureus* and *P. aeruginosa*, listed in Table SI2 in the Supporting Information. The bacterial coverage measured for each of these materials along with glass and the silver containing hydrogel as controls is shown in Figure SI3 in the Supporting Information.

Strains 40-1, 94-2 and TS-10 (Supporting Information, Table SI2) were excluded from further analysis as they were poor biofilm formers and therefore did not provide a stringent comparison. In all other cases the bacterial coverage on lead formulations was compared with silver hydrogels (Figure 2c–e). Bacterial coverage was reduced on all materials for the *S. aureus* strains compared with the silver containing hydrogel (Figure 2c). Significant reductions in bacterial coverage were observed for each of the *P. aeruginosa* strains on at least one lead formulation, however, large increases in coverage were also observed on some lead compositions compared with the silver hydrogel (Figure 2d). Thus, for the strains used in this study broad spectrum resistance to *S. aureus* strains was more readily achieved than with *P. aeruginosa.* It is important to note that the lead materials were not selected using these clinical strains. Thus the low coverage of these pathogens observed for the lead materials is indicative of the broad spectrum resistance to bacterial attachment achieved by this class of materials. However, before clinical implementation materials must be assessed for the absence of cytotoxic effects, which is the subject of ongoing in vitro and in vivo assessment. The material that resisted the attachment of the *S. aureus* strains best was the homopolymer of monomer 15 with an average reduction compared to the commercial silver containing hydrogel of 94% and up to 99% reduction for strain TS-4. This material was selected because of the low attachment of laboratory-adapted *S. aureus* strain 8325-4 to this material. The material with the lowest overall reduction of *P. aeruginosa* was produced from monomers 11 and 4 (4:1) with an average reduction of bacterial coverage compared to silver containing hydrogel of 36% and up to 86% reduction for strain 92-2. The greatest overall reduction of bacterial coverage for both strains was also observed on this material with an average reduction compared to silver containing hydrogel of 58%. This material was selected as a lead formulation due to its ability to resist multiple bacterial strains.

The photopolymerization method used in this study has been used on an industrial scale to produce coatings on a number of different materials for various applications, for example wire and cable coatings or vinyl flooring, thus may be suitable for producing coatings on medical devices.^18^ For a more ready route to low cost manufacturing, solution deposition of pre-synthesized polymer may be beneficial. For this, solution polymerization of the hit monomers can be undertaken to form linear polymers, something we have previously shown to be readily achievable whilst retaining the anti-attachment performance in the majority of hits.^12^

The ability of the lead formulations to prevent biofilm formation is achieved through resistance to bacterial attachment rather than through a killing mechanism. This is supported by the unaltered growth profile of bacteria in contact with hit materials^12^ and the successful culture of delicate embryonic stem cell lines on materials containing the hit monomers.^16^, ^19^ Established physicochemical mechanisms such as steric repulsion (de Gennes) and strong hydration^20^ appear less likely to be relevant to these materials based on their structural differences to oligoethylene glycols^7^ and relatively high water contact angles when compared with hydrogels, although detailed modeling investigations will be required to verify this. Thus, the lead formulations are likely a part of a new class of bacteria attachment resistant materials where the ester group combined with cyclic or aromatic hydrocarbon goups act together to resist bacterial attachment. In contrast, polystyrene, which contains a pendant benzene but no ester group, supports bacterial attachment and biofilm formation.^12^ It is currently unclear whether the mechanism of bacterial resistance of this class of weakly amphiphilic materials is physicochemical (e.g., preferential water binding as proposed for zwitterionic materials), or a result of molecular recognition of these surface structures and decision making by the bacteria. Bacterial recognition of the surface is thought possible because these polymers have been observed to resist the attachment of both Gram-positive *S. aureus* and Gram-negative *P. aeruginosa*, bacteria with very different biomolecular surface compositions, suggesting that the ability of bacterial cells to sense and respond to their immediate environment could be paramount. This may be a consequence of the individual cells or the bacterial population collectively recognising the nature of the polymer surface via cell envelope associated sensory proteins and/or through quorum sensing (bacterial cell-to-cell communication) mechanisms^21^ such that the lack of bacterial attachment occurs through these decision making processes.

In summary, we report a new class of materials resistant to bacterial attachment discovered using a high throughput discovery methodology with up to 81%, 99% and 99% reduction in bacterial coverage for *P aeruginosa*, *S. aureus* and UPEC, respectively, compared to a market leading anti-bacterial silver hydrogel. “Hit” materials were identified from over 600 unique materials and over 10 000 assays covering a broad cominatorial space. Furthermore, the “hit” materials were found to be resistant to the attachment of clinically isolated strains, which were outside the strains used for the high throughput screening process, demonstrating the potential clinical relevance of the lead compositions for reducing medical device associated infection.

## Experimental Section

*Polymer Array Synthesis*: Polymer microarrays were synthesized using the methods previously described.^14^, ^22^ Briefly, polymer microarrays were formed using a XYZ3200 dispensing station (Biodot) and metal pins (946MP6B, Arrayit). The printing conditions were O_2_ <1300 ppm, 25 °C, and 35% humidity. Polymerization solution was composed of monomer (75% v/v) in dimethyl formamide with photoinitiator 2,2-dimethoxy-2-phenylacetophenone (1% w/v). Monomers were purchased from Aldrich, Scientific Polymers and Polysciences and printed onto epoxy-coated slides (Xenopore) dip-coated with pHEMA (4% w/v, Sigma) in ethanol.

Polymer coupons were formed by pipeting polymerization solution (6 μL) onto a pHEMA coated slide and irradiating for 10 mins at O_2_ < 1300 ppm with a long wave UV source. Once formed polymers were dried at <50 mTorr for 7 days. Polymers were characterized by water contact angle measurements and time-of-flight secondary ion mass spectometry as previously described.^22^–^24^

*Cell Culture*: Three laboratory-adapted bacteria strains, *P. aeruginosa* PAO1, *S. aureus* 8325-4 and UPEC and clinical *P. aeruginosa* and *S. aureus* isolates were routinely grown on either LB (Luria-Bertani, Oxoid, UK) agar plates at 37 °C or in broth at 37 °C with 200 rpm shaking. Three GFP constitutively expressing plasmids, pME6032-GFP, pSB2019 and pSB2020^25^ were transformed into PAO1, 8325-4 and UPEC respectively and maintained in the bacteria by adding appropriate antibiotics to the culture media.

Slides were washed in distilled H_2_O for 10 min and air-dried before inoculation and growth of the bacteria under similar conditions as previously described.^26^–^27^ Briefly, UV-sterilized polymer slides were incubated in RPMI-1640 defined medium (15 mL, Aldrich) inoculated with diluted (OD_600_ = 0.01) GFP-tagged bacteria from overnight cultures at 37 °C with 60 rpm shaking for 72 h. The slides were removed from bacterial cultures and washed with phosphate buffered saline (PBS, 15 mL) at room temperature three times for 5 min each, then rinsed with distilled H_2_O and air dried. Fluorescence was measured using a GenePix Autoloader 4200AL Scanner (Molecular Devices, US) with a 488 nm excitation laser and a standard blue emission filter (510–560 nm) and processed using GenePix Pro 6 software (Molecular Devices, US). A similar bacterial attachment assay was also applied to scaled-up coupons. After washing with distilled H_2_O, the coupons were stained with SYTO17 dye (20 μM, Invitrogen, UK) at room temperature for 30 min. After air drying, the samples were examined using a Carl Zeiss LSM 700 Laser Scanning Microscope with ZEN 2009 imaging software (Carl Zeiss, Germany). The coverage of bacteria on the surface was analyzed using open source Image J 1.44 software (National Institute of Health, US).

The bacterial performance (*í*) was determined using Equation 1 where the subscript to the fluorescence signal (*F)* indicates the bacterial strain and *F*_max_ is the maximum fluorescence signal measured on any spot on the array for a given strain.

The *F* from each bacterial strain was determined using Equation 2 where *F*_test_ is the fluorescence intensity measured per unit area by the laser scanner after incubation with bacteria and *F*_control_ is the fluorescence intensity measured per unit area by the laser scanner measured on a control slide consisting of a replica array that was incubated in media for 72 h without bacteria. A limit of detection was applied to the data, such that if *F* was less than three times the standard deviation of a measurement it was given a value of zero.


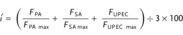
2

## Supporting Information

Supporting Information is available from the Wiley Online Library or from the author.

## Supplementary material

As a service to our authors and readers, this journal provides supporting information supplied by the authors. Such materials are peer reviewed and may be re-organized for online delivery, but are not copy-edited or typeset. Technical support issues arising from supporting information (other than missing files) should be addressed to the authors.
