# Supplementary material for: Discovery of Novel Materials with Broad Resistance to Bacterial Attachment Using Combinatorial Polymer Microarrays
Source: Adv Mater. 2013 Feb 18;25(18):2542–7. doi: 10.1002/adma.201204936 (PMC3736217; doi:10.1002/adma.201204936)
Supplement: Supplementary file 1 [file adma0025-2542-SD1.pdf]

Copyright WILEY-VCH Verlag GmbH & Co. KGaA, 69469 Weinheim, Germany, 2013.

ADVANCED MATERIALS

# Supporting Information

for *Adv. Mater.*, DOI: 10.1002/adma.201204936

# Discovery of Novel Materials with Broad Resistance to Bacterial Attachment Using Combinatorial Polymer Microarrays

*Andrew L. Hook, Chien-Yi Chang, Jing Yang, Steve Atkinson, Robert Langer, Daniel G. Anderson, Martyn C. Davies, Paul Williams, and Morgan R. Alexander**

# Discovery of novel materials with broad resistance to bacterial attachment using combinatorial polymer microarrays – Supplementary information

*By* Andrew L. Hook, Chien-Yi Chang, Jing Yang, Steve Atkinson, Robert Langer, Daniel G. Anderson, Martyn C. Davies, Paul Williams, *and* Morgan R. Alexander

**Table SI1.** A complete list of *F* for *P. aeruginosa* (PA), *S. aureus* (SA) and UPEC on 116 homopolymers.

| Monomer name | CAS number | $F_{PA}$ | $F_{SA}$ | $F_{UPEC}$ |
|---|---|---|---|---|
| Poly(propylene glycol) diacrylate | 52496-08-9 | 754429.5 | 618577.25 | 824092 |
| Hydroxypropyl methacrylate | 27813-02-1 | 433914.5 | 711057.75 | 0 |
| Glycidyl methacrylate | 106-91-2 | 1741128.75 | 1782278 | 2714390.25 |
| Tetrahydrofurfuryl methacrylate | 2455-24-5 | 0 | 103008 | 0 |
| Di(ethylene glycol) ethyl ether acrylate | 7328-17-8 | 21966911.25 | 1654367.25 | 1433283.25 |
| Ethyl acrylate | 140-88-5 | 1012726.75 | 766487.5 | 0 |
| Butylamino carbonyl oxy ethyl acrylate | 63225-53-6 | 6237787.75 | 715478.25 | 0 |
| Ethylene glycol diacrylate | 2274-11-5 | 109084.25 | 0 | 498060.75 |
| Butoxyethyl methacrylate | 13532-94-0 | 1714099.5 | 314256.5 | 66308.5 |
| Methyl 3-hydroxy-2-methylenebutyrate | 18020-65-0 | 1766908.5 | 2938696.75 | 5149233.25 |
| Lauryl methacrylate | 142-90-5 | 909323.25 | 363109 | 0 |
| Ethylene glycol dicyclopentenenyl ether acrylate | 65983-31-5 | 0 | 0 | 0 |
| Hexafluoroisopropyl acrylate | 2160-89-6 | 0 | 0 | 0 |
| Bisphenol A glycerolate diacrylate | 4687-94-9 | 0 | 0 | 0 |
| Hexadecafluoro-9-(trifluoromethyl)decyl acrylate | 15577-26-1 | 0 | 0 | 555656.5 |
| Butyl methacrylate | 97-88-1 | 916898.75 | 0 | 0 |
| Pentafluoropropyl methacrylate | 45115-53-5 | 775170.25 | 0 | 2064009.25 |
| Ethylhexyl methacrylate | 688-84-6 | 904171.5 | 98102 | 138594.75 |
| Tert-butylcyclohexylacrylate | 84100-23-2 | 0 | 0 | 0 |
| Trimethylolpropane ethoxylate triacrylate | 28961-43-5 | 0 | 0 | 7047425.75 |
| Tribromophenyl methacrylate | 37721-71-4 | 107533.5 | 0 | 68788.75 |
| Ethylhexyl acrylate | 103-11-7 | 0 | 0 | 521716 |
| Dodecafluoroheptyl acrylate | 2993-85-3 | 440423.5 | 0 | 0 |
| Glycidyl acrylate | | 0 | 16379370.5 | 19791401.25 |
| Ethylene glycol phenyl ether methacrylate | 10595-06-9 | 0 | 0 | 0 |
| Ethylene glycol methyl ether methacrylate | 6976-93-8 | 2244432 | 168695.5 | 134959 |
| Isobutyl acrylate | 106-63-8 | 1718564 | 0 | 0 |
| Butanediol diacrylate | 1070-70-8 | 190340.25 | 0 | 304926.75 |
| Bisphenol A propoxylate diacrylate | 67952-50-5 | 5607400.25 | 0 | 0 |
| Hexanediol ethoxylate diacrylate | | 0 | 350850.75 | 894851.5 |
| Hydroxybutyl methacrylate | 29008-35-3 | 369537.75 | 99599.5 | 0 |
| Methyl methacrylate | 35233-69-3 | 907155.25 | 0 | 928746 |
| Octafluoropentyl methacrylate | 355-93-1 | 614640.5 | 141157.5 | 0 |
| Ethoxyethyl acrylate | 106-74-1 | 11317679.25 | 419539 | 553358 |
| Butyl acrylate | 141-32-2 | 800073.5 | 0 | 0 |
| Tricyclodecane-dimethanol diacrylate | 42594-17-2 | 38699380 | 0 | 8907386.5 |
| Dimethylamino-propyl acrylate | 18526-07-3 | 25452568.75 | 0 | 0 |
| Hydroxyethyl methacrylate | 868-77-9 | 1751339.5 | 1224119 | 0 |
| Heptadecafluorodecyl methacrylate | 1996-88-9 | 1306635.75 | 0 | 242799.25 |
| Cyclohexyl methacrylate | 101-43-9 | 0 | 0 | 0 |
| Diethylamino ethyl acrylate | 2426-54-2 | 1992917.25 | 2416031.5 | 0 |
| Neopentyl glycol diacrylate | 2223-82-7 | 0 | 0 | 0 |
| Trimethylolpropane benzoate diacrylate | 79720-88-0 | 51865256.25 | 0 | 25900022 |
| Octafluoropentyl acrylate | 376-84-1 | 1165353.25 | 0 | 0 |
| Dimethylamino-ethyl methacrylate | 2867-47-2 | 8060612.5 | 3456221.25 | 1512849 |
| Isodecyl methacrylate | 29964-84-9 | 0 | 0 | 0 |
| Norbornyl methacrylate | 29753-02-4 | 0 | 0 | 0 |
| Isobornyl acrylate | 5888-33-5 | 68441.5 | 0 | 21997.25 |
| Neopentyl glycol propoxylate diacrylate | 84170-74-1 | 96317 | 125316 | 0 |
| Heptadecafluorodecyl acrylate | 27905-45-9 | 435058.75 | 63671.25 | 0 |
| Chloro-2-hydroxy-propyl methacrylate | 13159-52-9 | 0 | 0 | 0 |
| Hydroxybutyl acrylate | 2478-10-6 | 10417065.75 | 3802876.5 | 0 |
| Benzyl 2-n-propyl acrylate | 118785-93-6 | 1477152.25 | 359933.5 | 0 |
| Ethylene glycol dicyclopentenenyl ether methacrylate | 68586-19-6 | 0 | 0 | 304428.25 |
| Trimethylcyclohexyl methacrylate | 7779-31-9 | 0 | 0 | 0 |
| Hexyl acrylate | 2499-95-8 | 739271.5 | 0 | 0 |
| Methyl-1,2-ethanediyl bis[oxy(methyl-2,1-ethanediyl)]diacrylate | 42978-66-5 | 183188.5 | 0 | 235104.25 |

**Table SI1 continued**

| Monomer name | CAS number | $F_{PA}$ | $F_{SA}$ | $F_{UPEC}$ |
|---|---|---|---|---|
| Dipentaerythritol penta/hexa-acrylate | 60506-81-2 | 43415355.75 | 0 | 58059977 |
| Hexafluorobutyl acrylate | 54052-90-3 | 0 | 0 | 1701424 |
| Di(ethylene glycol) methyl ether methacrylate | 45103-58-0 | 9216204.75 | 530792.75 | 0 |
| Decyl methacrylate | 3179-47-3 | 351097.5 | 183753 | 0 |
| Hexafluoroisopropyl methacrylate | 3063-94-3 | 1637504.75 | 0 | 0 |
| Di(ethylene glycol) 2-ethylhexyl ether acrylate | 117646-83-0 | 4093941.5 | 370736.5 | 223306.75 |
| Trimethylhexyl acrylate | 2664-55-3 | 0 | 0 | 0 |
| Hexadecafluoro-2-hydroxy-10-(trifluoromethyl)undecyl methacrylate | 88752-37-8 | 0 | 0 | 538505.75 |
| Tridecafluorooctyl acrylate | 17527-29-6 | 0 | 0 | 0 |
| Isobutyl methacrylate | 97-86-9 | 1185691.25 | 0 | 0 |
| Dodecafluoro-7-(trifluoromethyl)-octyl acrylate | 50836-65-2 | 0 | 0 | 0 |
| Isobornyl methacrylate | 7534-94-3 | 0 | 0 | 0 |
| Isooctyl acrylate | 29590-42-9 | 2693980.5 | 392728.5 | 20692547.25 |
| Ethylene glycol methyl ether acrylate | 3121-61-7 | 3953855 | 0 | 0 |
| Trimethylolpropane triacrylate | 15625-89-5 | 222450.5 | 0 | 0 |
| Tridecafluoro-2-hydroxynonyl acrylate | 127377-12-2 | 0 | 0 | 0 |
| Allyl methacrylate | 96-05-9 | 0 | 0 | 0 |
| Hexyl methacrylate | 142-09-6 | 966920.75 | 267395.25 | 0 |
| Furfuryl methacrylate | 3454-28-2 | 1753791.5 | 0 | 0 |
| Tert-butyl acrylate | 1663-39-4 | 0 | 0 | 0 |
| Di(ethylene glycol) diacrylate | 4074-88-8 | 211690.75 | 0 | 1644977.5 |
| Trimethylolpropane propoxylate triacrylate | 53879-54-2 | 127742.75 | 34733.25 | 377753.25 |
| Trimethylolpropane ethoxylate methyl ether diacrylate | 302911-84-8 | 0 | 1320333.75 | 2121488.25 |
| Dimethylamino-ethyl acrylate | 2439-35-2 | 2334129.5 | 0 | 155051.5 |
| Ethyl 2-ethylacrylate | 3070-65-3 | 2479766.5 | 1614326 | 1384790.75 |
| Trimethylsilyloxy)ethyl methacrylate | 17407-09-9 | 4294533.5 | 3093099 | 1861807.5 |
| Tris(trimethylsilyloxy)-silyl propyl methacrylate | 17096-07-0 | 4753286.75 | 2541511.75 | 1432574.5 |
| Propargyl acrylate | 10477-47-1 | 299912 | 0 | 1541442.75 |
| Phosphoric acid 2-hydroxyethyl methacrylate ester | 52628-03-2 | 31395036 | 4025800.75 | 4008903.25 |
| Pentaerythritol triacrylate | 3524-68-3 | 54435981.75 | 0 | 45707543.25 |
| Hexafluoropent-1,5-diyl diacrylate | | 0 | 0 | 179989 |
| Ethoxyethyl methacrylate | 2370-63-0 | 2200640 | 258512.25 | 0 |
| Vinyl methacrylate | 4245-37-8 | 1688333.75 | 0 | 266091.5 |
| Heptafluorobutyl methacrylate | 13695-31-3 | 1493673.75 | 770116 | 0 |
| Ethylene glycol phenyl ether acrylate | 48145-04-6 | 219987 | 317283.75 | 576887.75 |
| Tetrahydrofurfuryl acrylate | 2399-48-6 | 15725596.5 | 2671600 | 1833627.5 |
| Bisphenol A propoxylate glycerolate diacrylate | 105650-05-3 | 0 | 0 | 1095889 |
| Methyl acrylate | 96-33-3 | 1463499.25 | 0 | 0 |
| Ter-butyl methacrylate | 585-07-9 | 1254612.25 | 0 | 91615.75 |
| Tridecafluorooctyl methacrylate | 2144-53-8 | 0 | 0 | 0 |
| Methylthioethyl methacrylate | 14216-23-0 | 586413.25 | 0 | 0 |
| Isodecyl acrylate | 1330-61-6 | 0 | 0 | 0 |
| Tetra(ethylene glycol) diacrylate | 17831-71-9 | 45421976.25 | 0 | 0 |
| Glycerol 1,3-diglycerolate diacrylate | 60453-84-1 | 6956927.25 | 1113226.25 | 0 |
| Pentafluoropropyl acrylate | 356-86-5 | 0 | 1596591.5 | 0 |
| Ethyl methacrylate | 97-63-2 | 0 | 0 | 0 |
| Diethylaminoethyl methacrylate | 105-16-8 | 3831227.75 | 639972.5 | 0 |
| Phenyl methacrylate | 2177-70-0 | 782439.5 | 509511.75 | 0 |
| Lauryl acrylate | 2156-97-0 | 1664645 | 0 | 0 |
| Hexamethylene diacrylate | 13048-33-4 | 137956.5 | 0 | 0 |
| Epoxidized acrylate | 91722-14-4 | 0 | 287844 | 648727.5 |
| Poly(propylene glycol) methyl ether acrylate | 83844-54-6 | 72901982 | 1036268.5 | 1154413.75 |
| Perfluorodecyl acrylate | 27905-45-9 | 0 | 0 | 0 |
| Ethyl 2-propylacrylate | 3550-06-9 | 881380.25 | 0 | 637083.25 |
| Benzyl methacrylate | 2495-37-6 | 0 | 0 | 0 |
| Zirconium carboxyethyl acrylate | 123633-53-4 | 11429964.25 | 3030234.5 | 17686331.25 |
| Heptafluorobutyl acrylate | 424-64-6 | 5257760.75 | 0 | 0 |
| Bisphenol F ethoxylate diacrylate | 120750-67-6 | 0 | 0 | 956104.75 |
| Poly(propylene glycol) acrylate | 50858-51-0 | 15668694 | 991637.25 | 1410742.75 |

**Table SI2.** List of bacterial strains isolated from clinical infections.

| Strain number | Strain | Source | Hit material | % reduction in bacterial coverage |
|---|---|---|---|---|
| TS-3 | *S. aureus* | Perineal wound | 10 (100%) | 98.0 |
| TS-4 | *S. aureus* | Recurrent boils | 10 (100%) | 98.0 |
| TS-10 | *S. aureus* | Abdominal wound | NA | NA |
| TS-11 | *S. aureus* | Fistula infection | 11:4 (4:1) | 99.7 |
| 40-1 | *P. aeruginosa* | Wound | NA | NA |
| 81-2 | *P. aeruginosa* | Wound | 10 (100%) | 79.1 |
| 82-2 | *P. aeruginosa* | Wound | 7:6 (4:1) | 95.2 |
| 92-2 | *P. aeruginosa* | Wound | 11:4 (4:1) | 98.0 |
| 94-2 | *P. aeruginosa* | Wound | NA | NA |

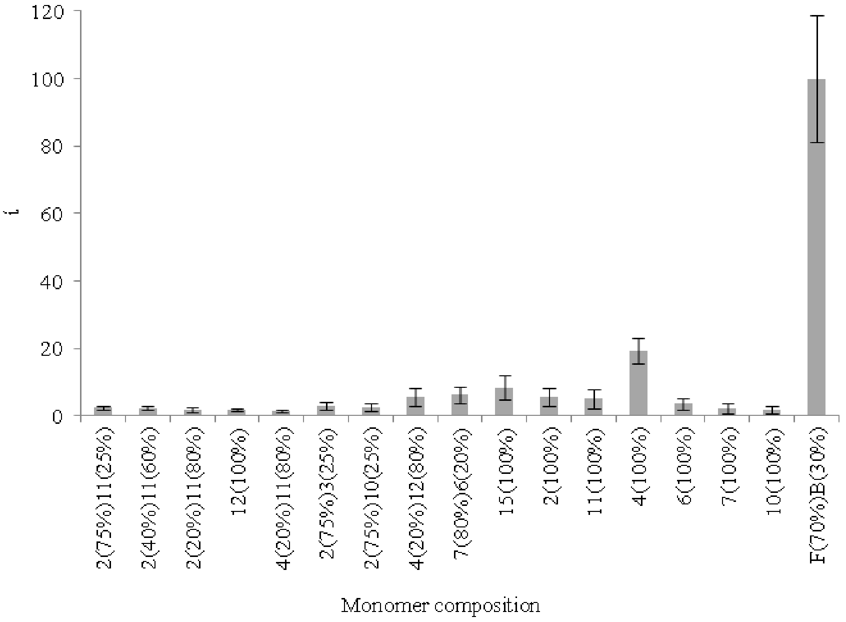


**Figure SI1.** Material scale out: ί for each of the hit formulations. The monomer numbers given refers to the monomers in Figure 1b(i). The error bars equal ± one standard deviation unit, n = 60.

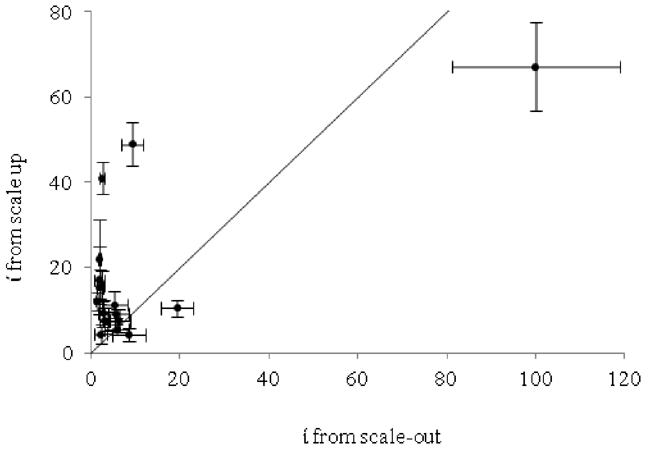


**Figure SI2.** Comparison of the biological performance of scaled up and scaled out samples. Error bars equal ± one standard deviation units, n = 3 for scale-up and whilst n = 60 for scale out. The y=x line is drawn as a guide.[1]

[1] A significantly different biological performance was observed for 4 of the 18 materials studied after scale up, specifically copolymers neopentyl glycol diacrylate (2) (75%) and *tert*-butylcyclohexyl acrylate (11) (25%), 2(40%)11(60%), 2(20%)11(80%), 4(20%)11(80%) and the homopolymers 12 (Figure SI2). It should be noted that some of the variance between the two assays is caused by the different staining procedures used. On the microarray all bacteria are labeled with GFP, thus the fluorescence signal will be representative of the total number of bacteria. On the polymer coupons the SYTO17 dye will stain both intracellular and extracellular DNA. Furthermore, the quantification is of coverage rather than total fluorescence, thus 3D structures will increase the value of the former measurement in comparison with the latter measurement. Despite this limitation, we have previously shown the two assays are comparable.[11] All the copolymers with varied performance contain monomer 11, however, the homopolymer of monomer 11 showed a relatively small change in performance between the two assays, with an ί of 5.3% ± 2.8% on the microarray and 11.2% ± 3.1% as a polymer coupon. We have previously observed changes in the surface chemistry of polymeric materials after scale up due to the surface enrichment of a specific pendant group due to the altered surface area to volume ratio that occurs upon scale up. This effect is more pronounced in copolymers where the two components may phase separate or surface segregate.[11]

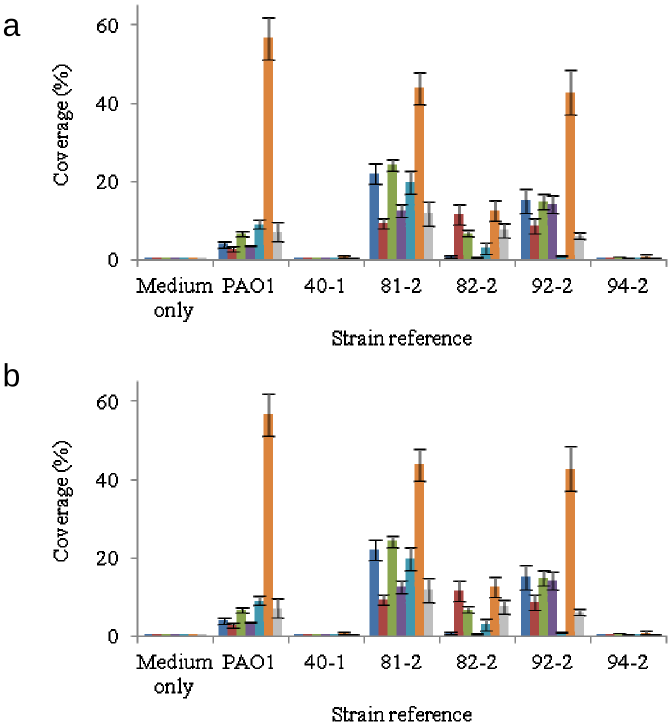


**Figure SI3.** Bacterial coverage of clinical isolates on scaled up hits. The composition of materials studied was monomers 2 (100%, ■), 15 (100%, ■), 7 (100%, ■), 7:6 (4:1, ■), 11:4 (4:1, ■), glass (■), silver hydrogel (■). The materials were cultured with (a) *P. aeruginosa* or (b) *S. aureus* strains. Error bars equal ± one standard deviation unit, n = 5.
